# Targeting CDC25C, PLK1 and CHEK1 to overcome Docetaxel resistance induced by loss of LZTS1 in prostate cancer

**DOI:** 10.18632/oncotarget.1574

**Published:** 2014-01-06

**Authors:** Nader Al Nakouzi, Sophie Cotteret, Frédéric Commo, Catherine Gaudin, Shanna Rajpar, Philippe Dessen, Philippe Vielh, Karim Fizazi, Anne Chauchereau

**Affiliations:** ^1^ Prostate Cancer Group, INSERM U981, Gustave Roussy, Villejuif, F-94805, France; ^2^ INSERM U981, LabEx LERMIT, Gustave Roussy; ^3^ INSERM U985, Gustave Roussy; ^4^ Department of Pathology, HistoCytoPathology Unit, Translational Research Laboratory and Biobank, Gustave Roussy; ^5^ Department of Medicine, Gustave Roussy; ^6^ University Paris-Sud 11, France

**Keywords:** Prostate cancer, chemoresistance, LZTS1, Cdc25c, PLK1, CHEK1

## Abstract

Docetaxel is used as a standard treatment in patients with metastatic castration-resistant prostate cancer. However, a large subset of patients develops resistance. Understanding resistance mechanisms, which are largely unknown, will allow identification of predictive biomarkers and therapeutic targets. We established resistant IGR-CaP1 prostate cancer cell lines for different doses of Docetaxel. We investigated gene expression profiles by microarray analyses in these cell lines and generated a signature of 99 highly differentially expressed genes potentially implicated in chemoresistance. We focused on the role of the cell cycle regulator *LZTS1*, which was under-expressed in the Docetaxel-resistant cell lines, its inhibition resulting from the promoter methylation. Knockdown of LZTS1 in parental cells with siRNA showed that LZTS1 plays a role in the acquisition of the resistant phenotype. Furthermore, we observed that targeting CDC25C, a partner of LZTS1, with the NSC663284 inhibitor specifically killed the Docetaxel-resistant cells. To further investigate the role of CDC25C, we used inhibitors of the mitotic kinases that regulate CDC25C. Inhibition of CHEK1 and PLK1 induced growth arrest and cell death in the resistant cells. Our findings identify an important role of LZTS1 through its regulation of CDC25C in Docetaxel resistance in prostate cancer and suggest that CDC25C, or the mitotic kinases CHEK1 and PLK1, could be efficient therapeutic targets to overcome Docetaxel resistance

## INTRODUCTION

Prostate cancer (PCa) is one of the most prevalent malignancies affecting men worldwide. It is the most frequent cancer in the United States and Western countries and is a major cause of cancer death and morbidity. In the past few years, clinical studies have highlighted the value of chemotherapy in metastatic PCa. Docetaxel-based chemotherapy benefit in castration-resistant prostate cancer (CRPC) was demonstrated in 2004 with an increase of overall survival [1,2] and it is until now the standard in first-line chemotherapy in CRPC. Docetaxel, a member of the taxane family, inhibits microtubules dynamics which triggers a G2/M cell cycle arrest of tumor cells, and induces apoptosis [3]. However, despite the survival benefit provided by this molecule, about half of patients develop drug resistance.

A pivotal strategy for the development of new drugs relies on the elucidation of resistance mechanisms and the identification of biological markers of response to Docetaxel to select patients who will benefit from taxane-based chemotherapy. Several studies highlighted the complex combination of gene expression enabling resistance to Docetaxel [4-6]. Domingo-Domenech et al. [7] showed that targeting Notch and HedgeHog pathways killed Docetaxel resistant cells using *in vivo* and *in vitro* models; Puhr et al. [8] showed that resistance is caused by epithelial-to-mesenchymal transition and loss of expression of miR-200c while miR-200b reverses Docetaxel resistance in lung adenocarcinoma [9].

We focused on the molecular mechanisms of Docetaxel resistance to identify relevant therapeutic targets to overcome this resistance. We developed a series of Docetaxel-resistant derivatives of the androgen-independent PCa cell line IGR-CaP1 [10] and performed a broad gene expression profiling using cDNA microarray analysis. We focused our efforts on the cell cycle regulator LZTS1, which is downregulated in our resistant model. The *LZTS1* gene was previously described as a tumor suppressor [11] and chromosomal deletions on chromosome 8p encompassing *LZTS1* are frequently observed in a variety of human cancers [12-16] including prostate cancer [17]. LZTS1 is a regulator of mitosis by maintaining high levels of CDC25C and CDK1 activity to prevent chromosomes missegregation [18]. Indeed, LZTS1 knockout results in accelerated mitotic progression, improper chromosome segregation and predisposes mice to cancer [18]. CDC25C plays an important role in mitosis by dephosphorylating CDK1 and allowing entry into mitosis. CDC25C is regulated by the checkpoint kinase 1 (CHEK1), which phosphorylates S216 and inactivates CDC25C, and by the Polo-like Kinase 1 (PLK1), which activates CDC25C by phosphorylating S198 and subsequently triggering activation of the CDK1/Cyclin B1 complex [19].

We used a siRNA knock-down strategy and a CDC25C inhibitor to investigate the role of LZTS1 and CDC25C in resistance to Docetaxel of IGR-CaP1 cells. To further demonstrate the role of CDC25C, we used pharmacological inhibitors of PLK1 and CHEK1, in our LZTS1-deficient Docetaxel resistant prostate cancer cells.

## RESULTS

### Establishment of Docetaxel-resistant cell lines

**Figure 1:**
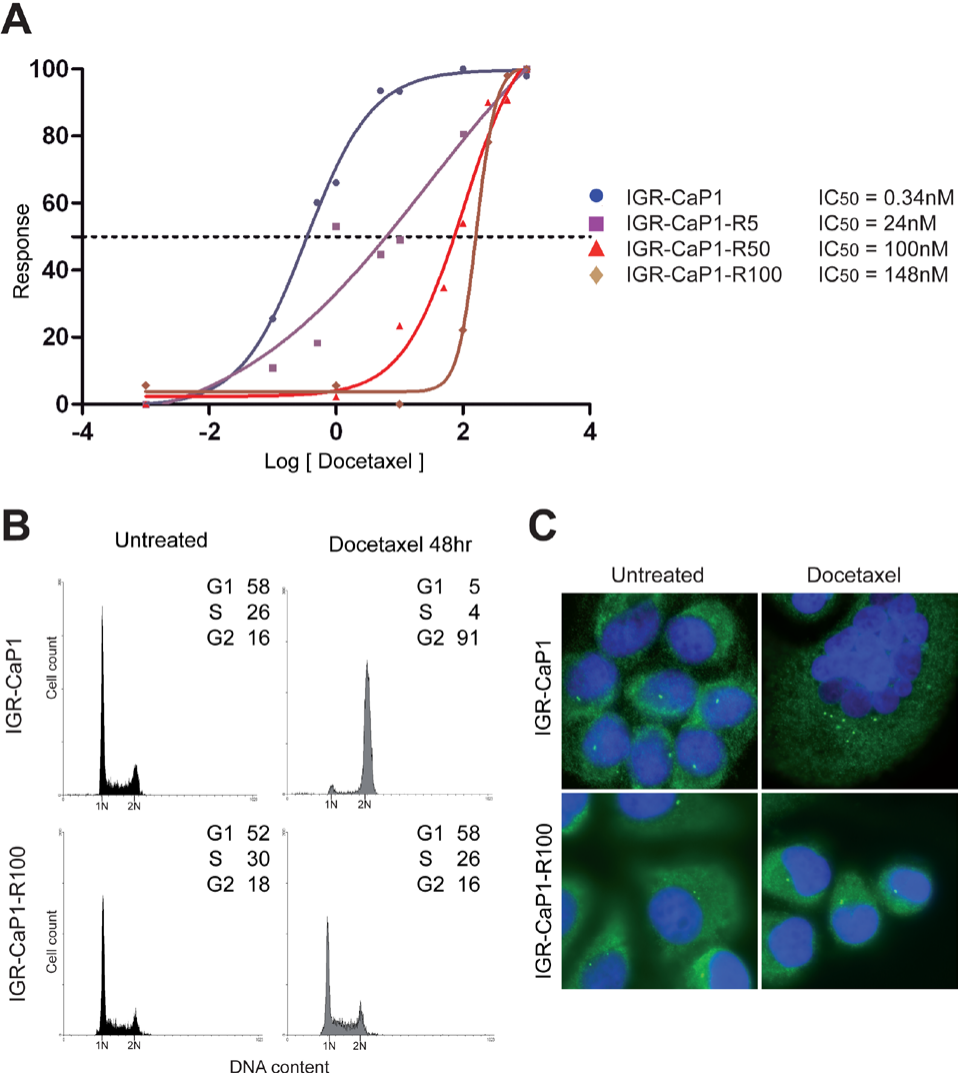
Characterization of Docetaxel-resistant cell lines A: Parental and resistant IGR-CaP1 cell lines were exposed to increasing concentrations of Docetaxel for 48h and cell survival was determined. Dose-response curves in IGR-CaP1-R5 (■) (IC_50_=24nM), IGR-CaP1-R50 (▲) (IC_50_=100nM) and IGR-CaP1-R100 cells (♦) (IC_50_=148nM) compared to parental IGR-CaP1 cells (●) (IC_50_=0.34nM). B: Representative cell cycle distributions of parental IGR-CaP1 and IGR-CaP1-R100 cells in the absence (untreated) or presence of 100nM of Docetaxel for 48h. X-axis: PI nucleic acid stain (DNA content); Y-axis: cell number per channel (counts). The percentage of cells in the different phase of the cycle is indicated. C: Immunofluorescence for γ-tubulin (green) showing the centrosomes. Nuclei were counterstained with Dapi (blue).

To generate a framework for studies of Docetaxel activity on PCa cells, we have developed six Docetaxel-resistant derivatives (IGR-CaP1-R5, -R12, -R25, -R50, -R100 and R200 respectively) of the IGR-CaP1 cell line [10], by periodically exposing proliferating cells to increasing doses of Docetaxel. Drug response of the parental IGR-CaP1 and Docetaxel-resistant IGR-CaP1-R cells was compared using a cell proliferation assay with increasing doses of Docetaxel. The IC_50_ value for the resistant cells increased from 24nM for IGR-CaP1-R5 cells to 148nM for IGR-CaP1-R100 compared to 0.34nM in parental cells, thus showing a ~400 fold higher level of Docetaxel resistance in IGR-CaP1-R100 compared to parental cells (Fig. 1A). The resistance of cells was confirmed by cell cycle analysis showing that, contrarily to IGR-CaP1, IGR-CaP1-R100 cells were not blocked in the G2/M phase (Fig. 1B). In IGR-CaP1 cells, Docetaxel induced cell death via mitotic catastrophe evidenced by profound multinucleation, polycentrosome and formation of giant cells (Fig. 1C). Importantly, in all the IGR-CaP1-R subclones, Docetaxel resistance was maintained in the presence of drug without inducing multinucleation, cell death, and a polycentrosome phenotype (Fig. 1C), suggesting that resistant cells have been able to generate mononucleated descendants by asymmetric cell division [20]. The IGR-CaP1-R100 cells grew more slowly than the parental cells (Fig. S1A), their growth rate being ~2 fold higher than that of the parental cells. Whereas cell survival assays showed that all IGR-CaP1 cells died after a 12nM-treatment with Docetaxel, IGR-CaP1-R100 cells were able to form colonies in the presence of Docetaxel (Fig. S1B).

### Inhibition of LZTS1 gene expression in Docetaxel-resistant IGR-CaP1-R cells

Microarray analysis was performed to compare expression profiles of genes in the six Docetaxel-resistant IGR-CaP1-R cell lines with parental cells. This analysis led to the identification of 244 probes associated with a resistant phenotype to all concentrations of Docetaxel (2D clustering with p-value<10^−10^, fold change >2). In this signature, 99 genes were strongly differentially expressed (fold change >5) in the resistant cells (Table SI). Validation of microarray data was confirmed by real-time qRT-PCR on 17 genes (Fig. S2). Based on the literature and Ingenuity^®^ Pathways analysis, we identified multiple pathways in our signature, highlighting the complex mechanisms mediating resistance to Docetaxel. We focused on cell cycle regulation and one of the genes, *LZTS1*, which has previously been described as a tumor suppressor and a cell cycle regulator [21], and investigated its functional role in the mechanisms of Docetaxel resistance.

**Figure 2:**
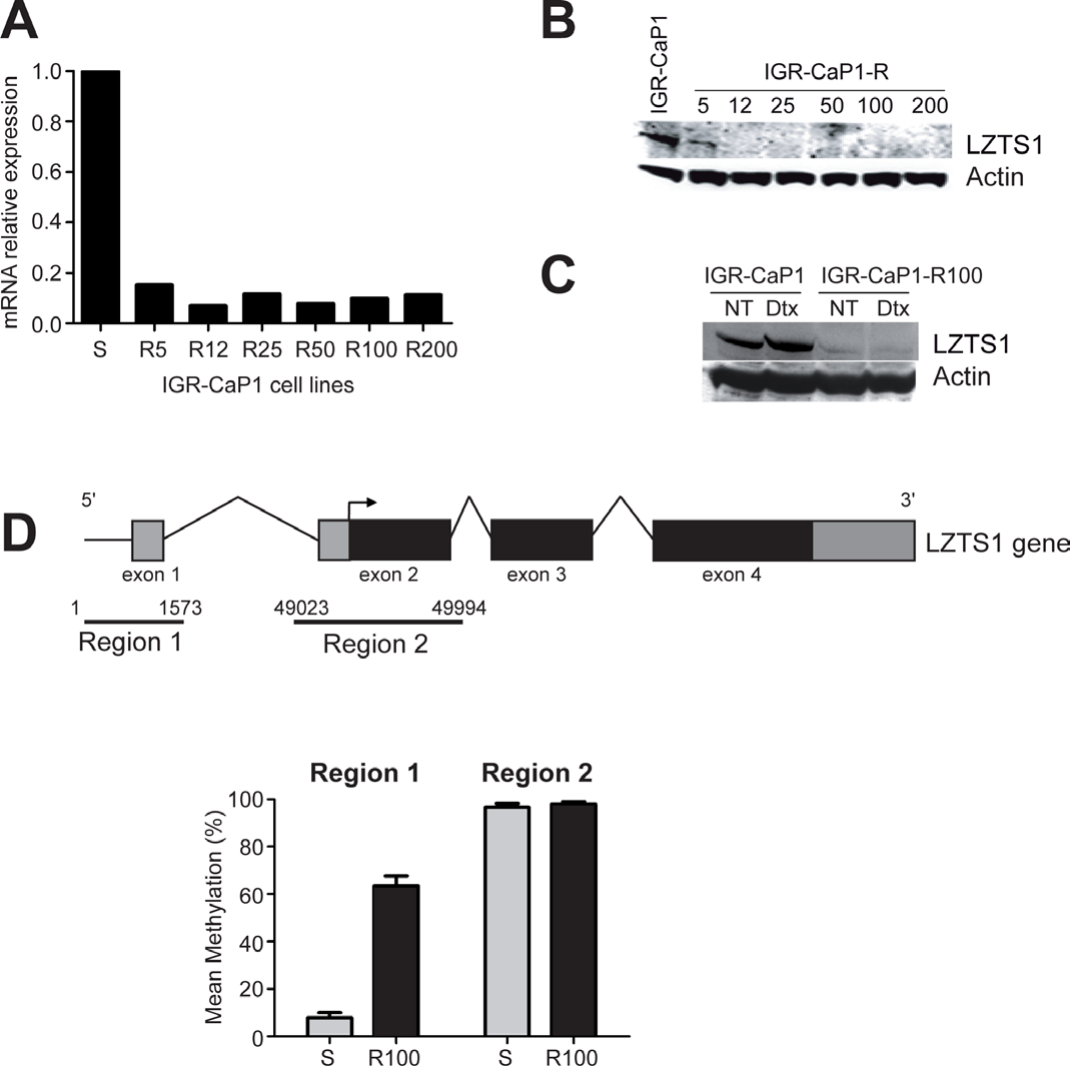
Inhibition of LZTS1 gene expression in Docetaxel-resistant IGR-CaP1-R cells A: Real-time qRT-PCR showing a high decrease of LZTS1 gene expression in the resistant cell lines. B: Whole cell extracts of parental and drug-resistant cells were subjected to immunoblotting with antibodies specific for LZTS1 or β-actin (loading control). C: Immunoblot realized as in B, after a 48h- treatment with 100nM of Docetaxel (Dtx) or without treatment (NT). D: Representative scheme of the larger form of the LZTS1 gene (LZTS1-001, total length 58999 nucleotides, transcript ID: ENST00000381569). The two regions of interest are indicated. Region 1 encompasses the sequence encoding exon 1 (601-824), region 2 encompasses the sequence encoding the exon 2 (49284-49727). Comparison of the mean methylation identified at 20 CpGs in region 1 and at 32 CpGs in region 2 was shown in parental IGR-CaP1 cells (S) and in Docetaxel-resistant (R100) cells.

*LZTS1* is down-regulated in resistant cells with a fold change of−6.0. We observed a high reduction in *LZTS1* mRNA levels by qRT-PCR in all the IGR-CaP1-R cells (80% of reduction) (Fig. 2A), which was correlated to a complete loss of protein expression (Fig. 2B). LZTS1 down-regulation was still observed at a 100nM-Docetaxel treatment for 48h (Fig. 2C) whereas Docetaxel had no effect on the expression levels of LZTS1 in the parental IGR-CaP-1 cells. Therefore, loss of expression of LZTS1 seemed to correlate with the resistance phenotype in IGR-CaP1-R100 cells. Previous studies reported that LZTS1 is frequently down-regulated in several solid tumors [12-15], and hypermethylation of a CpG island in the *LZTS1* promoter is frequently observed in cell lines and tumors which could be responsible for the reduced expression of LZTS1 in breast cancer cells [12]. To test this hypothesis, we analyzed promoter methylation of *LZTS1* in parental and resistant IGR-CaP1 cells. We found a high increase in methylation levels on 20 CpGs located on the 5' region 1 encompassing the non-coding exon 1 in resistant cells compared to parental ones (Fig. 2D and Table SII) but no difference in the methylation status in the second exon surrounding the transcription start site (region 2) as previously described [12]. These results suggest that loss of LZTS1 expression in the Docetaxel-resistant cells results from methylation of its promoter.

### LZTS1 down-regulation enhances Docetaxel-resistance of IGR-CaP1 cells

**Figure 3:**
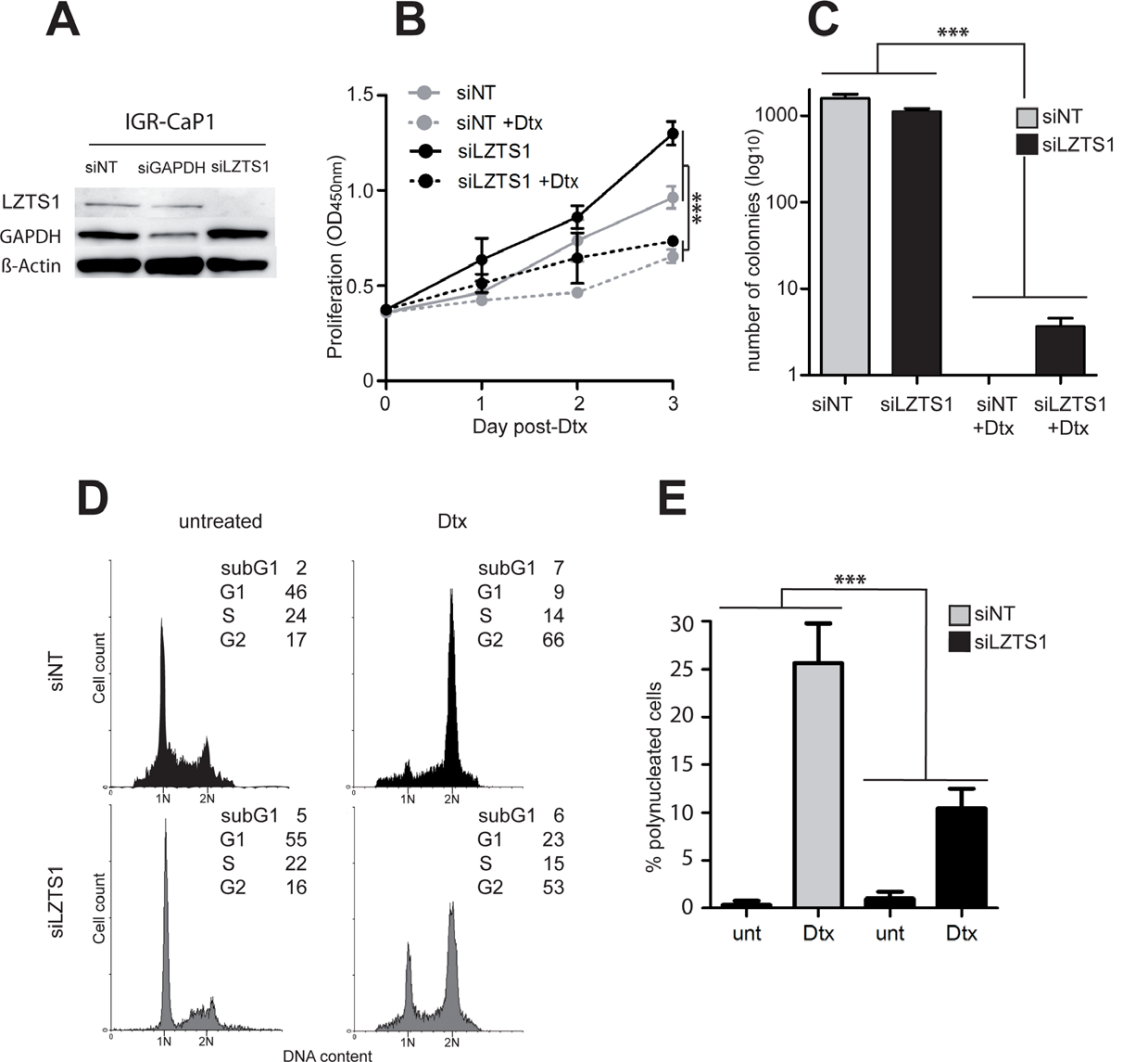
LZTS1 down-regulation enhances survival of IGR-CaP1 cells A: Inhibition of LZTS1 expression in IGR-CaP1 cells after a 48h-transfection with siRNA targeting LZTS1 (siLZTS1). Cells transfected with either a non-targeted siRNA (siNT) or a siRNA targeting GAPDH (siGAPDH) were used as control. Western-blot analysis was performed using specific antibodies for LZTS1, GAPDH or β-actin. These data are representative of three separate experiments. B: Growth curves comparing LZTS1-depleted cells (siLZTS1) with control IGR-CaP1 cells (siNT). 12nM of Docetaxel was added 24h after siRNA-transfection (hatched lines). C: Inhibition of LZTS1 reduced the number of IGR-CaP1 cell clones. D: Representative cell cycle distributions of LZTS1-depleted IGR-CaP1 cells (siLZTS1, grey) or in controls (siNT, black), in absence or presence of 100nM of Docetaxel as in Fig. 1 E: Analysis of polynucleation in LZTS1-depleted IGR-CaP1 cells or in controls in absence or presence of 12nM Docetaxel. In 3B, 3C and 3E, the two-way anova statistical analysis showed a significant interaction between siRNA and Dtx effects (***:*P*-value <0.001).

The functional role of LZTS1 in Docetaxel resistance was assessed in the parental IGR-CaP1 cells, using LZTS1-specific siRNA to mimic the loss of LZTS1 observed in the resistant cells. As shown in Fig. 3A, LZTS1 expression was entirely knocked down 48h after siLZTS1 transfection. Interestingly, growth curves showed that siLZTS1-transfected cells proliferated slightly more than the non-target siRNA-transfected cells (siNT) (Fig. 3B) with 12nM Docetaxel conditions, suggesting that LZTS1 extinction confers a growth advantage [22]. In a foci formation assay, the number of clones was similar in siLZTS1- compared to siNT-transfected cells in untreated cells, however, after a 12nM Docetaxel treatment, we still observed clones in siLZTS1-transfected cells (n=3.7 ±1.5) (Fig. 3C) whereas no clones were observed in control cells. Flow cytometry analyses showed that in LZTS1-knocked down cells, 100nM of Docetaxel led to a reduction in the G2/M blockage and an accumulation of cells in G1 (Fig. 3D). Furthermore, the number of polynucleated cells observed after treatment was significantly decreased in absence of LZTS1 (Fig. 3E). Together, these results showed that silencing of LZTS1 in parental IGR-CaP1 cells led to survival advantage, suggesting that LZTS1 is implicated in Docetaxel resistance.

### Role of the phosphatase CDC25C in the Docetaxel resistance mechanisms

**Figure 4:**
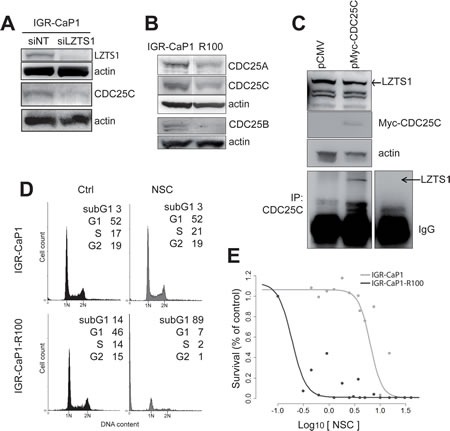
Implication of CDC25C in the Docetaxel resistance mechanism A: CDC25C expression is inhibited when LZTS1 is depleted. IGR-CaP1 cells were transfected with siRNA targeting LZTS1 or GAPDH or non-specific target. Whole cell extracts were subjected to immunoblotting using antibody specific for CDC25C. B: Expression of the three CDC25 phosphatases in parental and resistant cells. Whole cell extracts from IGR-CaP1 and IGR-CaP1-R100 were subjected to immunoblotting using antibodies for CDC25A, CDC25B, CDC25C. C: LZTS1 and CDC25C interaction. Cell extracts from IGR-CaP1 transfected with Myc-CDC25C were immunoprecipitated with anti-CDC25C and were subjected to immunoblotting using LZTS1 antibody. Actin was used as a loading control. D: Representative cell cycle distributions of IGR-CaP1 and IGR-CaP1-R100 cells in absence (Ctrl) or presence of 10µM NSC663284 inhibitor E: Dose-response curves showed a very significant difference in relative resistance to NSC663284 inhibitor in IGR-CaP1-R100 cells (black line) (IC_50_=0.2µM, range [0.15-0.28]) compared to IGR-CaP1 cells (grey line) (IC_50_=6.59µM, range [4.2-11]).

During mitosis, LZTS1 binds the CDK1 phosphatase CDC25C, which is then stabilized and protected from proteasomal degradation [18]. Partial or complete loss of LZTS1 downregulates CDC25C and inhibits CDK1 activity, leading to premature transition from metaphase to anaphase. We analyzed CDC25C expression levels in LZTS1-knocked down IGR-CaP1 and in LZTS1-deficient IGR-CaP1-R100 cells. As shown in Fig.4A, knock-down of LZTS1 in IGR-CaP1 cells induced a decrease in CDC25C and a decrease of CDC25C expression was also observed in IGR-CaP1-R100 cells (Fig. 4B). Noticeably, CDC25A and CDC25B phosphatases were also down-regulated in resistant cells. We further showed in IGR-CaP1 cells by co-immunoprecipitation that CDC25C interacts with LZTS1, as previously shown by Vecchione et al. [18] in 293 cells (Fig. 4C).

To further investigate the role of CDC25C in resistant cells, we treated IGR-CaP1 cells with the CDC25 phosphatases inhibitor NSC663284 [23,24]. Flow cytometry analysis showed that IGR-CaP1-R100 cells were much more sensitive to NSC663284, showing a massive cell death in NSC663284-treated cells (89%) compared to 3% in parental cells (Fig. 4D). The strong cytotoxic effect of NSC663284 on Docetaxel-resistant cells was confirmed by the NSC663284 dose-response curves which showed a ~33 fold lower IC_50_ in the IGR-CaP1-R100 cells compared to the parental cells (IC_50_=0.2µM vs 6.59µM) (Fig. 4E). Altogether, these results suggest that CDC25C plays a role in Docetaxel resistance and that CDC25C might be a therapeutic target to overcome Docetaxel resistance.

### Targeting the CDC25C-interacting kinase PLK1 to overcome Docetaxel resistance

All available CDC25C inhibitors exhibit mixed inhibition kinetics against CDC25A, CDC25B, and CDC25C. Therefore, we investigated the possibility of targeting the protein kinases that interact directly with CDC25C at the G2/M phase, PLK1 and CHEK1.

**Figure 5:**
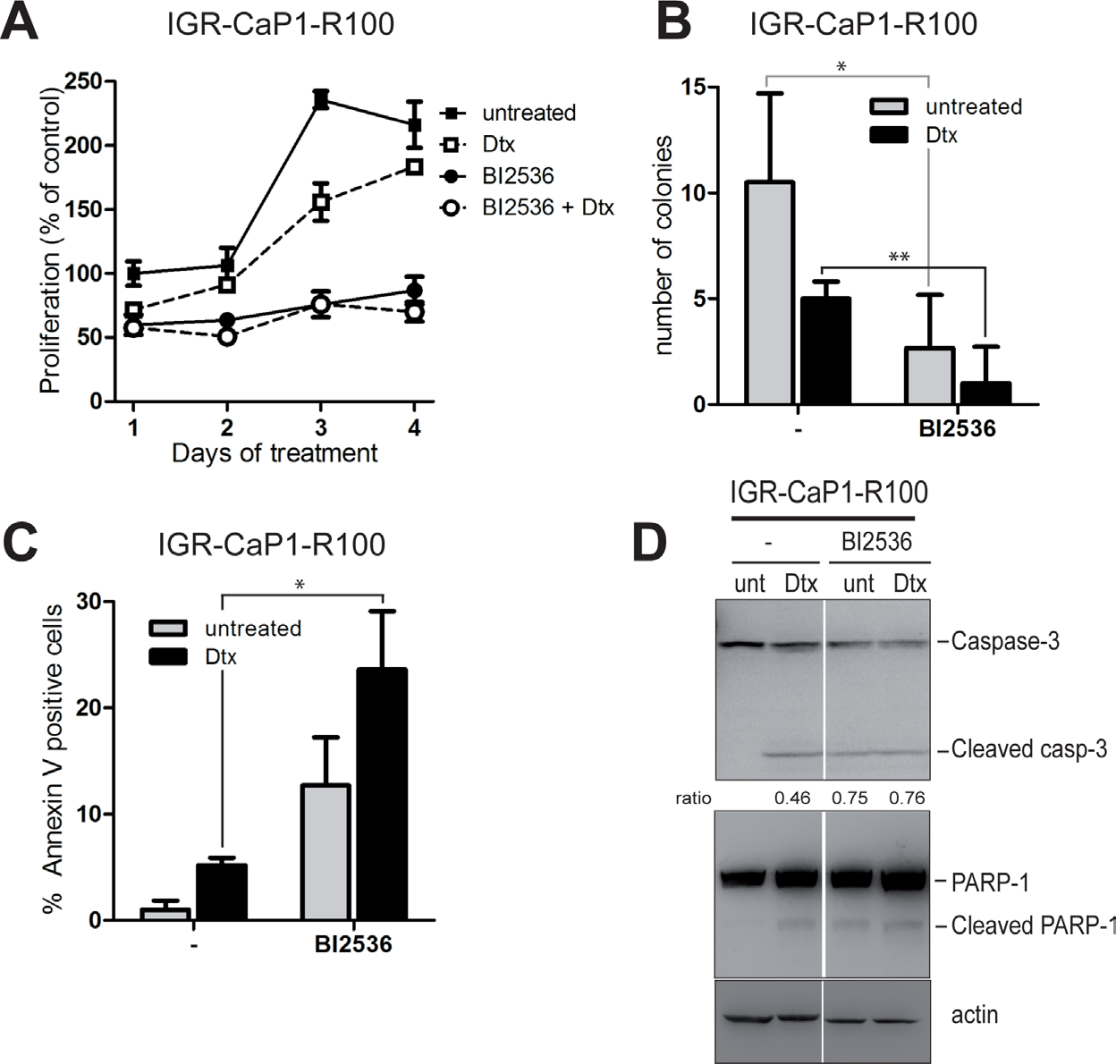
PLK1 inhibition induces cell death in Docetaxel-resistant cells A: Cell proliferation assay. IGR-CaP1-R100 cells were treated for 4 days with the PLK1 inhibitor BI2536 at 200nM in the absence or presence of Docetaxel and with Docetaxel alone (100nM). Cell growth was assessed every day using WST1. Data are represented as mean ± SEM. B: Colony formation assay. IGR-CaP1-R100 cells were seeded in 6-well plates in triplicate and treated with 200nM BI2536 in the absence or presence of Docetaxel and with Docetaxel alone (100nM). Cells were stained with crystal Violet 3 weeks later. Data are represented as mean ± SD. *P* value was derived from the two-tailed Student's *t* test, significantly different (*P<0.05,). C: Apoptosis. Cells were treated for 48h with 200nM BI2536 in the absence or presence of Docetaxel and with Docetaxel alone (100nM). Apoptosis was assessed using annexinV and propidium iodide staining. *P* value was derived from the two-tailed Student's *t* test, significantly different (*P<0.05,). D: PARP and Caspase-3 cleavage. Cells were treated as in (C). Western-blot analysis was performed using specific antibodies for PARP1, Caspase-3 or β-actin. Ratio of cleaved Caspase-3 vs full-length is indicated.

PLK1 activates CDC25C and Cyclin B1 by phosphorylation, which triggers entry of cells into mitosis, [25]. Interestingly, PLK1 and CDC25C are overexpressed in prostate cancer [26,27], and PLK1 expression correlates with high tumor grades [28]. We thus investigated the ability of several PLK1 inhibitors currently in clinical trials to induce apoptosis in the Docetaxel-resistant cells. We assessed the effects of three different PLK1 inhibitors [25], BI2536, BI6727 or TAK-960, on cell proliferation of parental and resistant IGR-CaP1 cells. BI6727 and TAK-960 reduced cell proliferation (data not shown) whereas BI2536 induced growth arrest in both parental (Fig. S3A) and resistant cells (Fig. 5A) when used alone and in combination with Docetaxel. To further study the effects of BI2536 on cell growth, we performed colony formation assays. Inhibition of PLK1 with BI2536 alone or with Docetaxel (Fig. 5B) strongly decreased the formation of colonies in resistant cells and abrogated colony formation in IGR-CaP1 cells (Fig. S3B). Targeting PLK1 has been shown to induce apoptosis in prostate cancer cells after radiation [29], therefore we investigated whether BI2536 could trigger apoptosis in our model. Annexin V stainings showed that PLK1 inhibition induced apoptosis in the Docetaxel-resistant cells (up to 30%) (Fig. 5C) and in the parental IGR-CaP1 cells (Fig. S3C), and was significant both with BI2536 alone and in combination with Docetaxel. As shown in Fig. 5D, BI2536-treated cells displayed cleaved PARP and Caspase-3 confirming induction of apoptosis.

### Targeting the CDC25C-interacting kinase CHEK1 to overcome Docetaxel resistance

**Figure 6:**
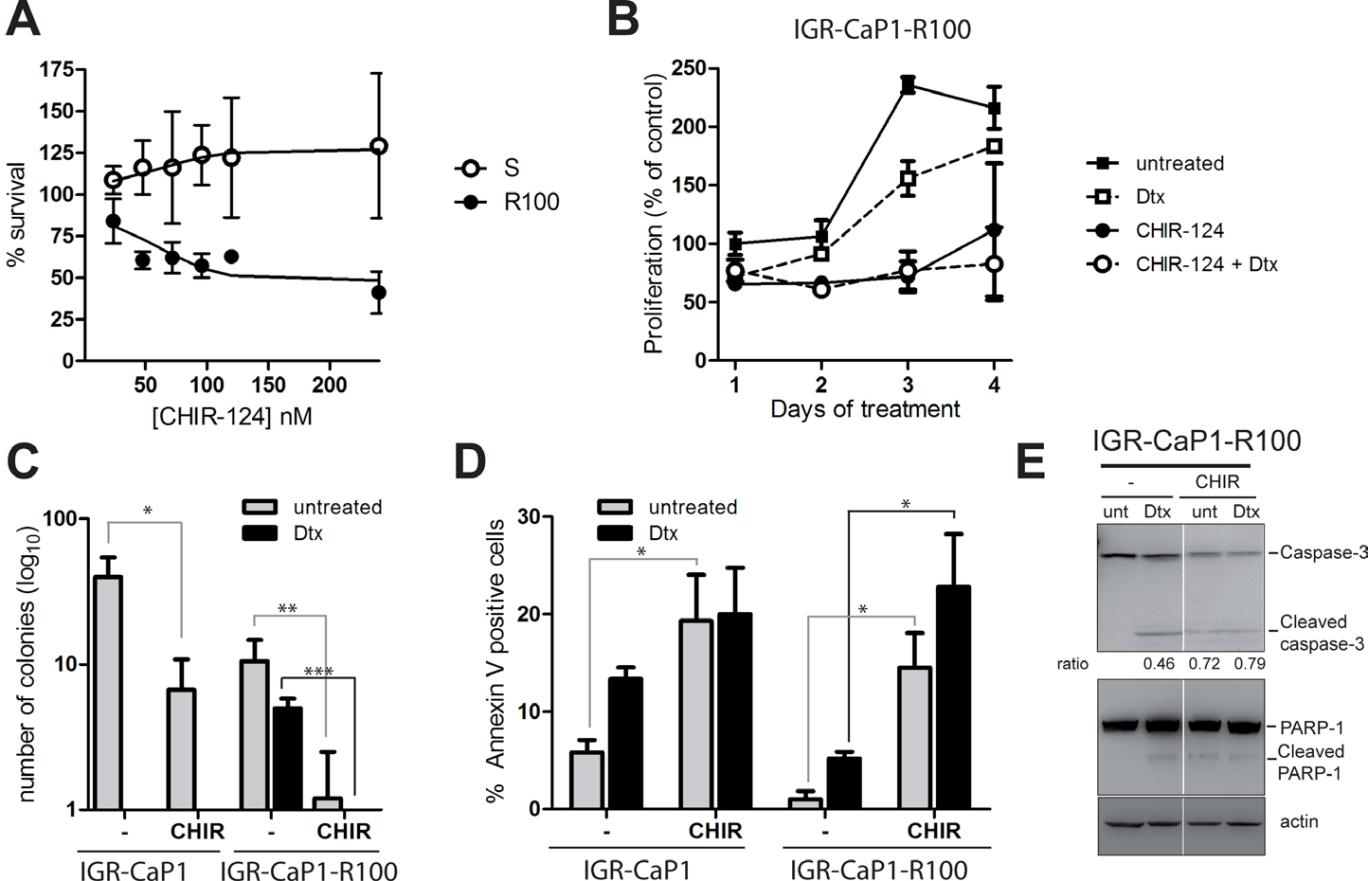
CHEK1 inhibition induces cell death in Docetaxel-resistant cells A: Dose-dependent growth of IGR-CaP1 and IGR-CaP1-R100 cells. Cells were treated with various concentrations of CHIR-124 for 48h. Cell proliferation was assessed with WST1. B: Cell proliferation of IGR-CaP1-R100 cells. Cells were treated with 100nM CHIR-124 in the presence or absence of 100nM Docetaxel or with Docetaxel alone during 4 days. Proliferation was assessed using WST1. C: Colony formation assay. IGR-CaP1-R100 cells were seeded in 6-well plates in triplicate and treated with CHIR-124 in the absence or presence of Docetaxel and with Docetaxel alone (100nM). Cells were stained with crystal Violet 3 weeks later. Data are represented as mean ± SD for >3 independent experiments. *P* value was derived from the two-tailed Student's *t* test, significantly different (*P<0.05, **P<0.01, ***P<0.001). D: Apoptosis. Cells were treated for 48h with 100nM CHIR-124 in the absence or presence of Docetaxel and with Docetaxel alone (100nM). Apoptosis was assessed using annexinV and propidium iodide staining. Data are represented as mean ± SEM. *P* value was derived from the two-tailed Student's *t* test, significantly different (*P<0.05). E: PARP and Caspase-3 cleavage. Cells were treated as in (C). Western-blot analysis was performed using specific antibodies for PARP1, Caspase-3 or β-actin. Ratio of cleaved Caspase-3 vs full-lenght is indicated.

Chek1 inhibits CDC25C through serine-216 phosphorylation causing a G2/M arrest in response to genotoxic stress [30,31]. We further examined the impact of targeting CHEK1 with the specific pharmacological inhibitor CHIR-124 [32] on the survival of the LZTS1-deficient Docetaxel-resistant cells. Fig. 6A shows a dose-dependent response of parental and resistant IGR-CaP1 cells to CHIR-124 after a 48hr-treatment. CHIR-124 impaired cell growth of resistant cells starting at 100nM but had no effect on IGR-CaP1 cells. We next investigated cell proliferation in the presence of 100nM CHIR-124 during 4 days, alone or with Docetaxel (Fig. 6B). CHIR-124 alone caused cell growth arrest until day 3 when cells started growing again. In contrast, cell proliferation was abolished when treated with Docetaxel and CHIR-124 at day 4. This effect was more pronounced in Docetaxel-resistant cells with a significant 30% decrease in survival than in the parental cells (16%) (Fig. S4A and S4B). Importantly, CHIR-124 treatment alone highly decreased the ability of cells to form colonies (83% decrease in parental cells vs 89% in resistant cells) and it totally abolished the ability of Docetaxel-resistant cells to form colonies when used in combination with Docetaxel (Fig. 6C). We next determined if CHIR-124 triggered apoptosis in our cells. As shown in Fig. 6D, CHIR-124 induced apoptosis in both parental and resistant cells and the combination with Docetaxel slightly increased the percentage of apoptotic cells. Apoptosis was confirmed by immunoblotting showing PARP-1 and Caspase-3 cleavage in treated cells (Fig. 6E). Altogether, our results show that the targeting of CHEK1 with CHIR-124 in combination with Docetaxel induces cell growth arrest and cell death of the Docetaxel-resistant cells.

## DISCUSSION

Docetaxel consistently improves survival of metastatic CRPC but resistance eventually occurs and the search for predictive biomarkers to select patients responding to the drug has been disappointing. A greater understanding of resistance pathways is needed to both predict resistance early in the course of treatment and to ultimately overcome this resistance and improve outcome. In the era of personalized cancer therapy, significant treatment advances have occurred through a better understanding of drug resistance and molecular heterogeneity among patients with the same disease.

To elucidate mechanisms of resistance to Docetaxel and to potentially *identify new* therapeutic *targets*, we established Docetaxel-resistant cells from the IGR-CaP1 cell line [10]. Comparison of transcript profiles between Docetaxel-resistant IGR-CaP1-R and parental cell lines revealed a robust modification in the transcription level of 99 genes. Among these genes, we identified the cell cycle regulator *LZTS1*. The cytotoxicity of taxane-based chemotherapy such as Docetaxel has previously been shown to occur in part through perturbation of the cell cycle and mitotic checkpoints [4] by disrupting microtubules depolymerization leading to cell death. In our study, we report that *LZTS1* is inhibited by methylation of its promoter in the resistant IGR-CaP1-R cells. Importantly, LZTS1 is present on chromosome 8p, and in many cancers, deletion of chromosome 8p12-22 or DNA hypermethylation of this region are associated with a more aggressive tumor phenotype, tumor progression and more rapid appearance of metastases [12-15]. We demonstrated that reducing LZTS1 expression levels with specific siRNA in the parental IGR-CaP1 cells allows the survival of several clones after Docetaxel treatment. This provides compelling evidence that loss of *LZTS1* expression is likely to be involved in Docetaxel resistance. LZTS1 has been shown to function as a mitotic regulator via its interaction with the mitotic kinase CDK1 in the G2/M phase of the cell cycle [18] and its regulation of the CDK1 phosphatase CDC25C. Anti-sense oligonucleotides against *LZTS1* reduce active CDK1 levels, which indicates that LZTS1 may stabilize the CDK1–cyclinB1 complex in G2/M. In this case, inactivation of LZTS1 leads to early exit from mitosis and reduced control of proliferation [21]. Furthermore, MEFs lacking LZTS1 display enhanced mitotic degradation of CDC25C, impaired CDK1 activation, accelerated mitotic progression and chromosomal instability [18]. Therefore, we suggest that, in absence of LZTS1 expression, tumor cells are able to bypass the checkpoint, are not arrested in mitosis and do not show features of mitotic catastrophe (Fig. 3D and 3E), suggesting that targeting the checkpoint might provide a mean to induce apoptosis in resistant cells.

Microarray data analysis show that many genes and pathways are involved in Docetaxel resistance highlighting the complexity of chemoresistance mechanisms, as evidenced by our results and observations made by others [5,7,33]. We report that LZTS1 depletion partially confers Docetaxel resistance by down-regulating CDC25C. CDC25C belongs to a family of three conserved dual specificity phosphatases, CDC25A, CDC25B, CDC25C, which regulate cyclin-dependent kinases. CDC25C is overexpressed in PCa in its non-phosphorylated active form [26,34], however, no studies have reported thus far any role for CDC25C in resistance to Docetaxel. In this study, we show that inhibition of CDC25C with a pharmacological inhibitor has a strong cytotoxic effect on Docetaxel-resistant cells, which was not observed in Docetaxel-sensitive counterparts suggesting that CDC25C might be a therapeutic target to overcome Docetaxel resistance. However, the NSC663284 inhibitor, which we used in our experiments, has also the ability to inhibit CDC25A and CDC25B, and we cannot exclude that these isoforms are also targeted. Moreover, CDC25C has not been druggable so far and specific inhibitors and *in vivo* active molecules have not yet been developed [35]. Our results suggest that in absence of LZTS1 expression, targeting CDC25C could very efficiently kill resistant cells, but because of these caveats, we decided to investigate if targeting the mitotic kinases regulating CDC25C, PLK1 and CHEK1, could be the proper approach to overcome Docetaxel resistance in our LZTS1-deficient cells.

CHEK1 phosphorylates CDC25C at S216 leading to its inactivation and PLK1 phosphorylates CDC25C at S198, which leads to its nuclear translocation and activation of CDK1. The implication of PLK1 in Docetaxel resistance was indeed recently shown in lung adenocarcinoma cells [36]. Targeting of PLK1 using the BI2536 inhibitor in combination with conventional chemotherapy was recently shown to impair tumor growth *in vivo* in two xenografts models established from biopsies of triple negative breast cancer patients [37]. In prostate cancer cells, inhibition of PLK1 with BI2536 significantly potentiated Paclitaxel-mediated cell death [38] while combination of BI2536 or BI6727 with histone deacetylases had antitumor effects *in vitro* [39]. Targeted inhibition of PLK1 causes mitotic catastrophe and induction of apoptosis in prostate cancer cells. Additionally, PLK1 is overexpressed in prostate tumors and its expression is correlated to higher tumor grades [28] suggesting that PLK1 might be a potential therapeutic target for prostate cancer [29,40]. Currently, PLK1 inhibitors are being evaluated in registered clinical trials, namely volasertib (BI-6727) which was reported to have clinical response and TAK-960, which possesses the highest specificity towards PLK1, and inhibits the growth of tumor cells *in vitro* and *in vivo* independently of MDR1 expression [41]. In our model, all PLK1 inhibitors worked in terms of cell growth arrest and apoptosis induction, but BI2536 gave the best results on cell growth arrest, as evidenced by the cell proliferation curves and the formation of colonies (Fig. 5A and 5B).

CHEK1 inhibitors have been tested with classical chemotherapeutic agents in other solid tumors and CHEK1 is considered a good target to increase the therapeutic effectiveness of anticancer agents [42]. Several CHEK1 inhibitors have been used in clinical trials in advanced solid tumors such as LY2603618 [43-45]. In particular, sensitization to Docetaxel with CHEK1 antagonist PF-004477736 has been observed in colon and breast cancer xenografts [46]. In our model, LY2603618 had only a slight effect on the resistant cells (data not shown), so we used another CHEK1 specific inhibitor, CHIR-124 [32]. We show that it induces apoptosis and cell growth arrest in the resistant cells. We also observed a potentiating effect of the combination of CHEK1 inhibitor with Docetaxel which was more pronounced in the Docetaxel-resistant cells. In our model, CHEK1 inhibitors are more efficient to induce cell growth arrest than the PLK1 inhibitors (Fig. 5B and 6C).

Overall, our results show a stronger effect of both CHEK1 and PLK1 inhibitors in combination with Docetaxel in resistant cells and this potentiating effect may be due to targeting the mitotic checkpoint which is bypassed when LZTS1 is inhibited. We also show that indirectly targeting CDC25C with inhibitors of CHEK1 and PLK1 provides a mean to overcome chemoresistance.

## MATERIALS AND METHODS

### Cell culture, selection of Docetaxel-resistant clones and reagents

The IGR-CaP1 cell line was maintained in RPMI1640 medium supplemented with 10% FBS. Docetaxel-resistant clones were selected by exposing cells to Docetaxel in a dose-escalation manner as described [33]. Surviving clones to low dose of Docetaxel were subsequently subjected to 5nM, 12nM, 25nM, 50nM, 100nM and 200nM of Docetaxel. Cells freely dividing in each dose of Docetaxel-containing media were considered resistant. Docetaxel (TAXOTERE^®^) was kindly provided by Sanofi-Aventis (France). NSC663284 was purchased from Calbiochem; BI2536 and CHIR-124 were purchased from Selleckchem and were resuspended in DMSO. Anti-LZTS1 (C-20), and anti-CDC25C (C-20) were obtained from Santa-Cruz Biotechnology, anti-CDC25A, anti-CDC25B, from Cell Signalling, anti-GAPDH and anti-β-actin from Sigma.

### Cell Cycle analysis

Cell cycle was determined using propidium iodide (PI) staining. Briefly, parental and Docetaxel-resistant cells were treated or not with Docetaxel for 48h and cells were collected by trypsinization. After staining with PI, cells were analyzed with a FACS Calibur cytometer (Becton Dickinson, Le Pont-De-Claix, France).

### Total RNA Preparation and Reverse Transcription

Total RNA from parental and Docetaxel-resistant cells was isolated using Trizol (Invitrogen) and purified with RNeasy Micro Kit (Qiagen). The RNA Integrity Number (RIN) was assessed on the Agilent 2100 Bioanalyzer device (Agilent Technologies, Massy, France). All specimens included in this study displayed a RIN of 10.

### Oligo Microarray Technology

Gene expression was profiled using a 4×44K Human Whole Genome (G4112F) expression array (Agilent Technologies, Santa Clara, Cal.) with a dye-swap competitive hybridization procedure, according to the manufacturer's instructions. Total RNA from untreated parental IGR-CaP1 cells was used as the RNA reference. Total RNA from IGR-CaP1 cells resistant to 5nM, 12nM, 25nM 50nM, 100nM, and 200nM of Docetaxel respectively, were used as samples. Image analyses (quantification, normalization) were performed with Feature Extraction software (Agilent Technologies) and gene expression analysis was performed using Resolver software (Rosetta Inpharmatics). Analysis of genes differentially expressed between parental and resistant cell lines was performed with an absolute fold change >2 and p-value <10^−10^. Using this procedure for each of the 6 combined experiments, a list of 244 probes was extracted. These genes were sorted out by the mean fold change obtained from the mean of Log(Ratio) for the 6 doses of resistance to Docetaxel. The list of the 99 mostly modified genes (fold change >5 and p-value <10^−10^) is shown in Table SI. All raw microarray data are available on Array Express at the European Bioinformatics Institute (http://www.ebi.ac.uk/arrayexpress; accession number: E-MTAB-1221).

### Quantitative real-time RT-PCR

Real-time RT-PCR was performed using the ABI Prism7900 System (Applied Biosystems - Life Technologies, Saint-Aubin, France) as described [10] with PCR primers for LZTS1 (Hs00232762_m1) (Applied Biosystems). The ∆∆CT method was used to quantify transcripts.

#### Genomic DNA extraction and bisulphite modification

One microgram of genomic DNA isolated using the QIAamp DNA mini kit (Qiagen) was bisulfite-modified using CpGenome DNA Modification Kit (Chemicon). DNA CpGenome Universal Methylated DNA (Chemicon) and human genomic DNA (Clontech) were used as positive and negative controls, respectively.

### Bisulfite Sequencing Analysis

Primers were designed to include methylated and unmethylated alleles overlapping 2 regions 1-1573 (1573bp) and 49023-49994 (971 bp) covering the exon 1 and 2 respectively of the LZTS1 gene. To analyse the CpG methylation status of LZTS1 promoter, PCR reaction was carried out in a 50μl mixture containing 0.2mM each dNTP, 1.5mM or 3mM MgCl2, 400nM of each primer, 80ng of bisulfite-treated DNA and 1U GoTaq Hot Start Polymerase (Promega). The PCR cycling profile consisted of a step at 95°C for 2min, followed by 4 cycles of 94°C for 30sec, 62°C for 30sec and 72°C for 30sec; 38 cycles of 94°C for 30sec, 60°C for 30sec and 72°C for 30 sec; and 72°C for 7min. PCR products were run in 1% agarose gel and were purified using Sephadex G-50 (Amersham Biosciences, Cleveland, OH, USA) and then directly sequenced using the BigDye Term v1.1 Cycle Sequencing Kit (Applied Biosystems). Sequencing reactions were purified enzymatically using ExoSAP-IT (Affimetrix, Santa Clara) and were run on an ABI 3730 automated sequencer (Applied Biosystems - Life Technologies, Saint-Aubin, France). The collected data were analysed using SeqScape analysis software (Applied Biosystems). The methylation status of CpG islands was determined by direct sequencing of both strands and by estimation of the relative peak height of the PCR products.

### siRNA transfection and Western blot analysis

Cells were transiently transfected with synthetic siRNA (Stealth RNAi™) targeting the genes of interest or negative controls (Invitrogen). Transfections were carried out using lipofectamine RNAiMax (Invitrogen). Forty hours or 72h after transfection, cell survival assays were performed on untreated cells or treated with Docetaxel as mentioned in figure legends. Whole cell extracts were prepared in RIPA buffer with proteases inhibitors (Roche) and 40µg of lysates were used for Western-Blot probed with specific antibodies. Immunoblot analyses were performed using the enhanced chemoluminescence-based detection kit (Pierce). For immunoprecipitation, IGR-CaP1 cells were transfected with the pCMV-CDC25C expression vector (Origene) or pCMV empty vector using jetPRIME transfection reagent (Ozyme). 48h after transfection, CDC25C and LZTS1 proteins were co-immunoprecipitated using standard procedures and subjected to immunoblotting. Cells were treated for 48h with inhibitors in the presence or absence of Docetaxel, lysed in RIPA buffer and subjected to immunoblotting.

### Cell viability assays

Cells were seeded into 96-well plates and treated with increasing Docetaxel concentrations for 48h. Cell viability was determined using the WST1 reagent (Roche). Cell viability in the treated plates was compared to untreated cells to calculate the surviving fraction. The dose-response curve and IC50 were then estimated with a weighted 4- or 5-parameters logistic regression, as previously described [47].

### Foci formation assays

One thousand–5,000 cells/plate were plated onto 10cm dishes 48h after siRNA transfections and prior to a 24h-docetaxel treatment. Cells were plated in 60mm dishes 48h prior treatment with 100 nM CHIR124 or 200nM BI2536 in the presence or absence of Docetaxel. Eight to 10 days later, plates were stained with Crystal violet (Sigma-Aldrich) and clones were counted.

### Immunofluorescence microscopy

Cells were plated on coverslips and grown for 2 days. For centrosome analysis, fixation was previously described [48]. Cells were labelled with anti-γ-tubulin (1:1000) (GTU-88, Sigma) antibody followed by incubation with AlexaFluor 488-conjugated antibody (Molecular Probes). Nuclei were fixed in 4% formaldehyde. Images were acquired on Zeiss Axioplan 2 microscope.

## Supplementary Figures and Tables




