# Supplementary material for: Targeting CDC25C, PLK1 and CHEK1 to overcome Docetaxel resistance induced by loss of LZTS1 in prostate cancer
Source: Oncotarget. 2014 Jan 6;5(3):667–78. doi: 10.18632/oncotarget.1574 (PMC3996665; doi:10.18632/oncotarget.1574)
Supplement: Supplementary file 1 [file oncotarget-05-0667-s001.pdf]

Targeting CDC25C, PLK1 and CHEK1 to overcome Docetaxel resistance induced by loss of LZTS1 in prostate cancer - AL NAKOUZI et al

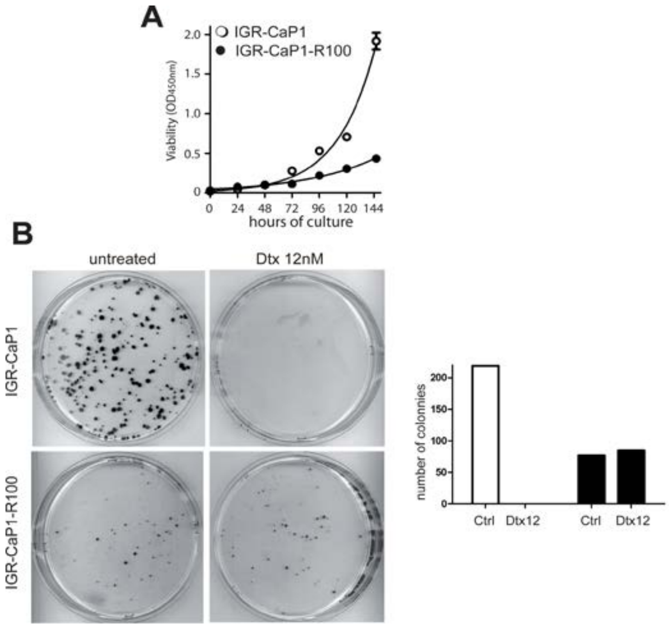

Figure S1: Cell-growth characteristics of Docetaxel-resistant cells
A: The growth rate of IGR-CaP1 (○) and Docetaxel-resistant IGR-CaP1-R100 cells (●) was determined as described in Materials and Methods. **B:** The cell survival assay was performed after 2 weeks of culture in presence or in absence of 12nM of Docetaxel. The number of clones was determined.

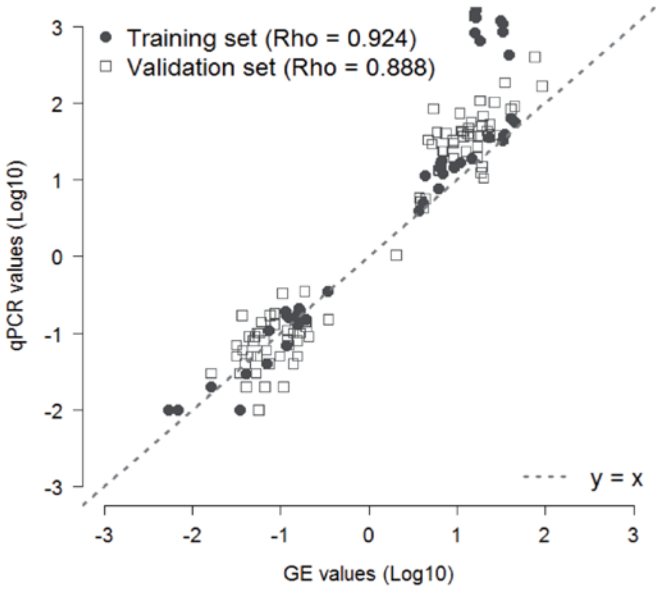

Figure S2: Validation of microarray data by real-time RT-PCR
A group of 17 genes found in the Docetaxel-resistance signature of 99 genes have been studied by quantitative RT-PCR. Comparison between microarray (GE values) and RT-PCR data by a Spearman test showed a very good correlation in the same samples used for microarray and RT-PCR (training set ●) (rho=0.924) and in a set of independent samples (validation set □) (rho=0.888).

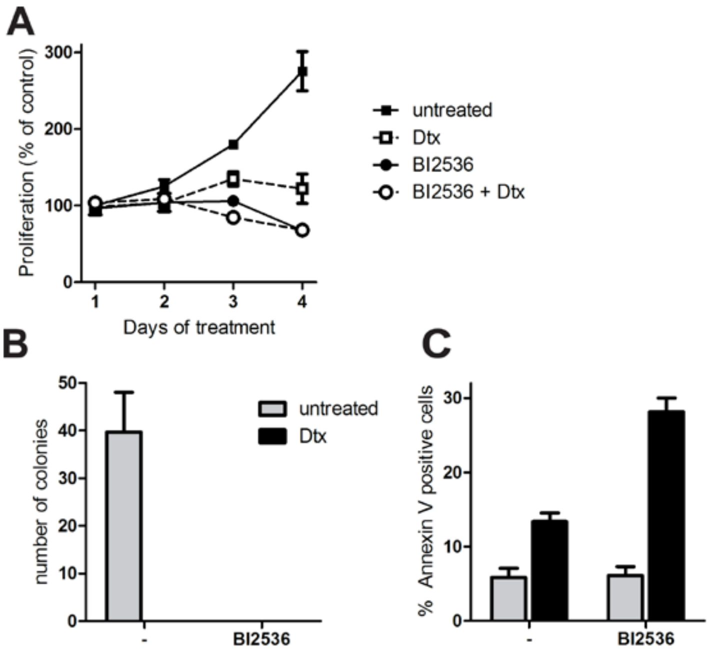

Figure S3: BI2536 enhances the cell death in parental IGR-CaP1 cells

A: Cell proliferation of IGR-CaP1 parental cells. Cells were treated with 200nM BI2536 (round symbols) in the presence or absence of 12nM Docetaxel or with Docetaxel alone during 4 days. Proliferation was assessed using WST1. Data are represented as mean ± SEM. **B:** Colony formation assay. IGR-CaP1 cells were seeded in 6-well plates in triplicate and treated with 200nM BI2536 in the absence or presence of Docetaxel and with 12nM Docetaxel alone. Cells were stained with crystal Violet 3 weeks later. Data are represented as mean ± SD. **C:** Apoptosis. IGR-CaP1 cells were treated for 48h with 200nM BI2536 in the presence or absence of 12nM Docetaxel or with Docetaxel alone. Apoptosis was assessed using annexinV and propidium iodide staining. Data are represented as mean ± SEM.

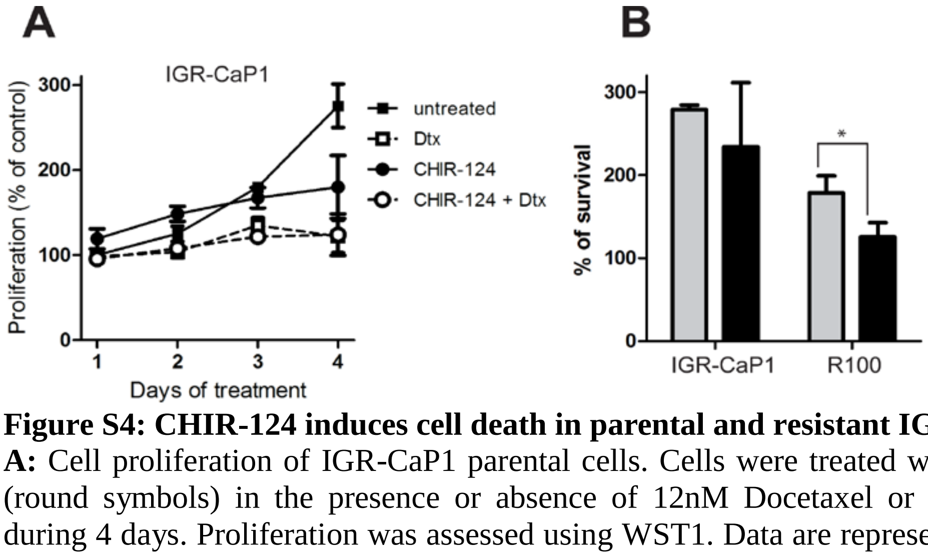

Figure S4: CHIR-124 induces cell death in parental and resistant IGR-CaP1 cells

A: Cell proliferation of IGR-CaP1 parental cells. Cells were treated with 100nM CHIR-124 (round symbols) in the presence or absence of 12nM Docetaxel or with Docetaxel alone during 4 days. Proliferation was assessed using WST1. Data are represented as mean ± SD of three independent experiments.**P*<0.05, P value was derived from the two-tailed Student's *t* test. **B:** Comparison of the inhibitory effect of CHIR-124 on cell viability between the parental and the resistant cells after 4 days of treatment. Data represented mean ± SD of three independent experiments. **P*<0.05, P value was derived from the two-tailed Student's *t* test.
